# Immune response to *Ichthyophthirius multifiliis* and role of IgT

**DOI:** 10.1111/pim.12675

**Published:** 2019-10-17

**Authors:** Kurt Buchmann

**Affiliations:** ^1^ Department of Veterinary and Animal Science Faculty of Health and Medical Sciences University of Copenhagen Frederiksberg C Denmark

**Keywords:** adaptive, fish, immunity, immunoglobulin, innate

## Abstract

The parasitic ciliate *Ichthyophthirius multifiliis* causes white spot disease in freshwater fish worldwide. The theront penetrates external surfaces of the naïve fish where it develops into the feeding trophont stage and elicits a protective immune response both at the affected site as well as at the systemic level. The present work compiles data and presents an overall model of the protective reactions induced. A wide spectrum of inflammatory reactions are established upon invasion but the specific protection is provided by adaptive factors. Immunoglobulin IgT is involved in protection of surfaces in several fish species and is thereby one of the first adaptive immune molecules reacting with the penetrating theront. IgT producing lymphocytes occur in epithelia, dispersed or associated with lymphoid cell aggregations (skin epidermis, fins, gills, nostrils and buccal cavities) but they are also present in central immune organs such as the head kidney, spleen and liver. When theronts invade immunized fish skin, they are encountered by host factors which opsonize the parasite and may result in complement activation, phagocytosis or cell‐mediated killing. However, antibody (IgT, IgM and IgD) binding to parasite cilia has been suggested to alter parasite behaviour and induce an escape reaction, whereby specific IgT (or other classes of immunoglobulin in fish surfaces) takes a central role in protection against the parasite.

## 1 INTRODUCTION

**Figure 1:**
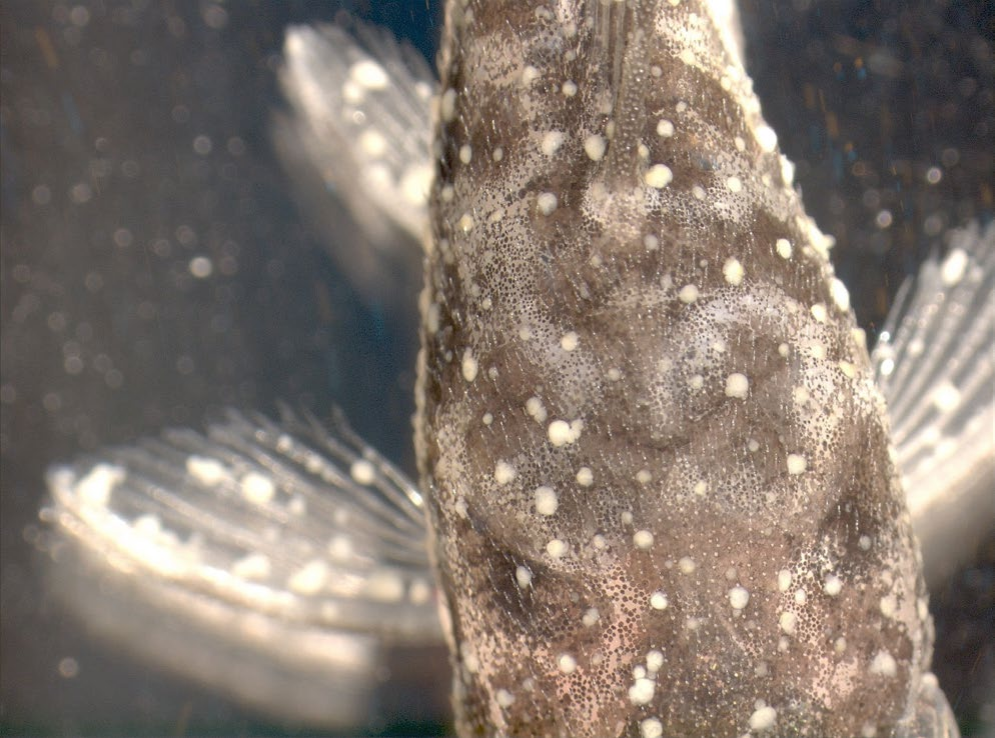
White spots containing *Ichthyophthirius multifiliis* trophonts (diameters 0.5‐1.0 mm) in teleost (*Ancistrus* sp) epidermis

The parasitic ciliate *Ichthyophthirius multifiliis* is known to infect a wide range of freshwater teleosts worldwide and elicit the disease ichthyophthiriosis.^1^ The pathognomonic white spots in the skin of the infected fish, which is the basis for the vernacular name of the disease, white spot disease WSD, are caused by the feeding stage of the parasite, the trophont, which induces proliferation of the epidermal cells enclosing the parasite. The continuously rotating ciliate in its epidermal enclosure appears as a light reflecting blister on the fish surface visible to the naked eye as a white spot (Figure 1). Heavy infections may be lethal but fish surviving an infection were already a century ago reported to be protected against reinfection.^2^ The protection is correlated to the severity of the primary infection,^3^ but the immunological mechanisms associated with the protection were largely unknown until it was demonstrated that immune carp produced substances in skin and plasma which were able to immobilize the infective stages (theronts) of the parasite.^4^ Subsequent investigations have shown that specific immunoglobulins may explain the immobilizing ability through cross‐linking i‐antigens on the parasite surface.^5^, ^6^ This stimulus may alter the behaviour of the ciliate, and induce an escape reaction.^7^ However, it is evident that various host cells (comprising lymphocytes and granulocytes) are involved in the immunization process.^8^, ^9^, ^10^, ^11^, ^12^, ^13^, ^14^ In addition, recent transcriptomic studies have demonstrated that a wide range of other immune factors are activated following infection.^15^, ^16^ This suggests that host protection is based on a more differentiated and complicated immune response than previously outlined.

### 1.1 Life cycle

**Figure 2:**
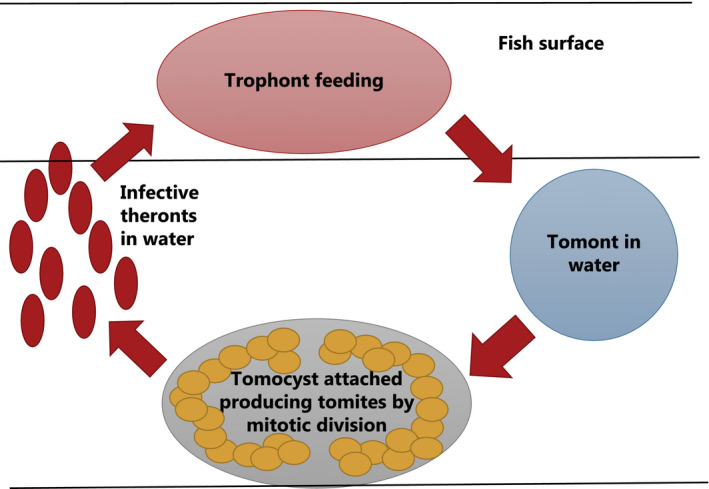
Schematic view of the life cycle stages of *Ichthyophthirius multifiliis*

**Figure 3:**
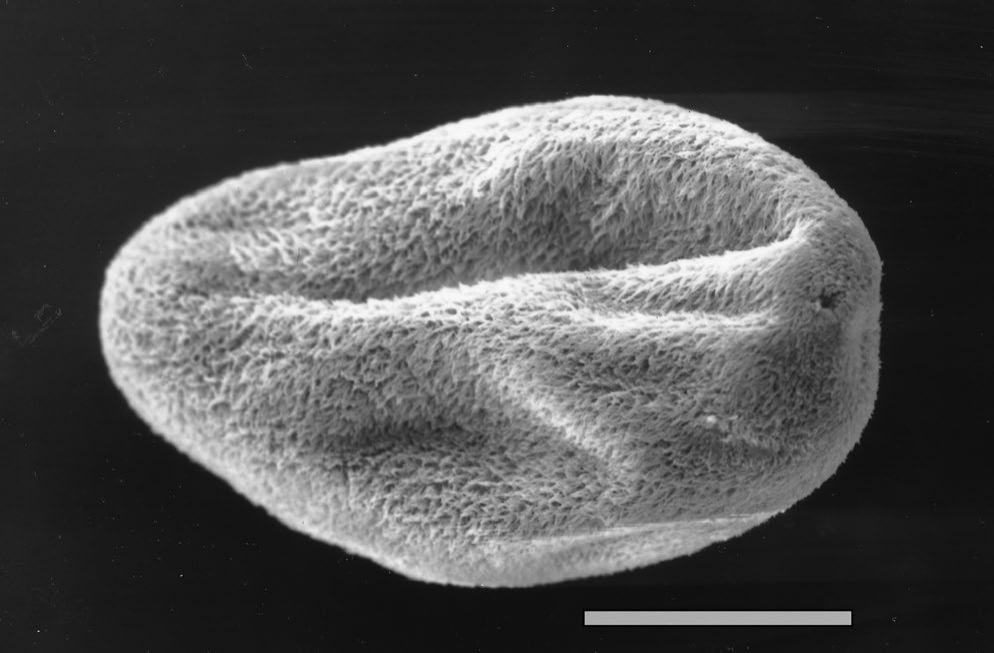
Trophont of *Ichthyophthirius multifiliis* escaped from trout skin epidermis attaining the tomont stage. Scale bar 100 µm. From^58^

**Figure 4:**
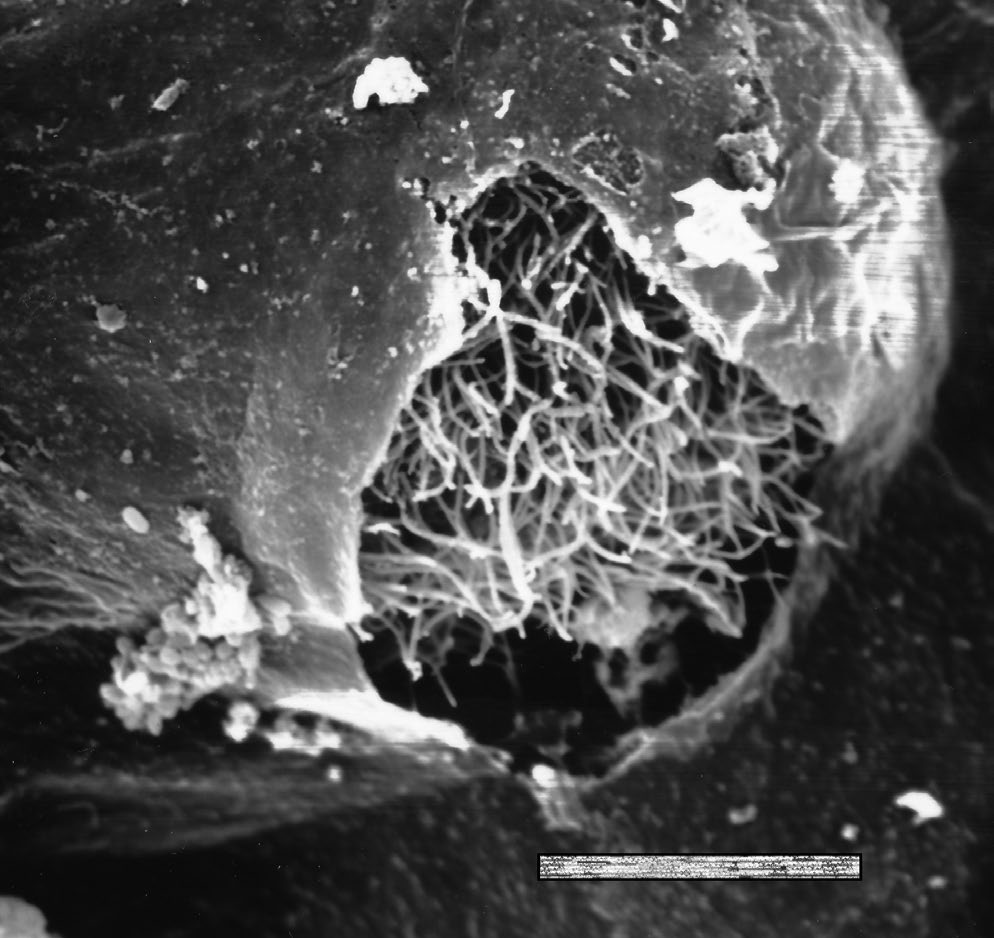
Opening of the tomocyst wall with appearing theront. Scale bar 10 µm. From^58^

**Figure 5:**
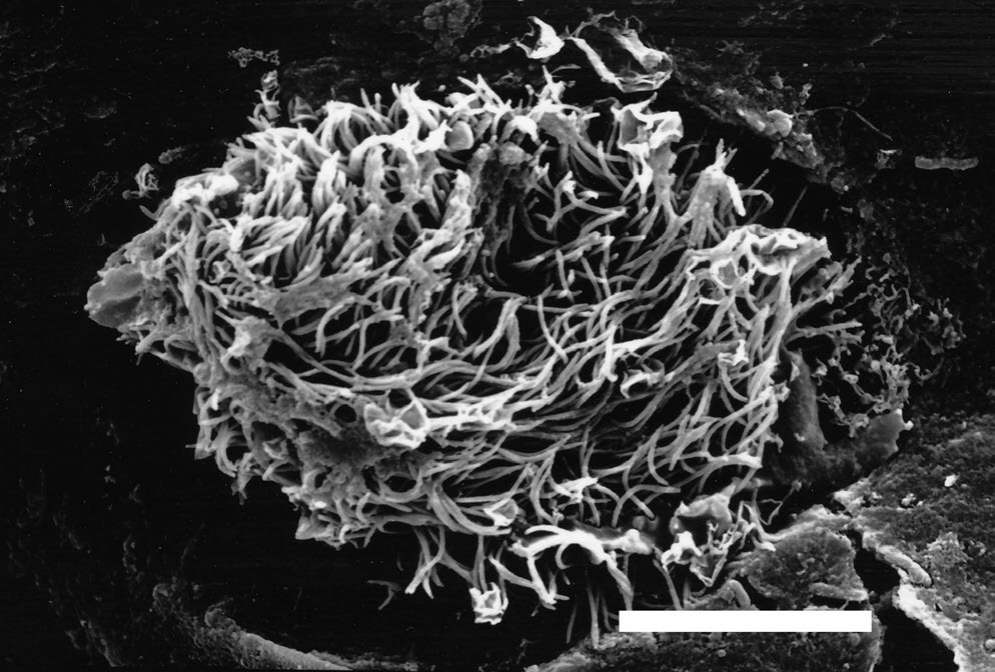
Infective theront released from the tomocyst. Scale bar 10 µm. From^58^

The life cycle of *I multifiliis* comprises four stages^17^, ^18^ (Figure 2). The feeding stage in the epidermis is termed the trophont, and it is richly equipped with cilia (Figure 3). When reaching a size of 0.1‐1.0 mm, it can break out of its infection focus and attain a new stage, termed the tomont, which actively (still by ciliary action) moves in water for minutes to hours before it settles on firm substrates (glass, plastic, wood, plants and fish tank wall). Here, it attains a tomocyst stage as it produces an external protective jelly‐like substance, whereafter it initiates a series of mitotic divisions resulting in several hundreds of tomites. These ciliated tomites move vividly inside the tomocyst and escape continuously through openings in the gelatinous coating (Figure 4). The liberated and free‐swimming ciliated cell, termed the theront, seeks and penetrates the fish host surface and attains the early trophont stage (Figure 5).

### 1.2 Protection

Protective immunity against the parasitic ciliate as previously described^2^, ^3^, ^4^ was later confirmed^1^, ^19^, ^20^ and inspired development of various prototype vaccines. Intraperitoneal injection of killed or live parasite stages conferred protection,^21^, ^22^, ^23^, ^24^, ^25^, ^26^, ^27^ but although in vitro procedures for cultivation of the parasite might be developed^28^ methods for bulk production of parasites for vaccines is not at hand. Recombinant protein antigen containing vaccines^29^, ^30^ and DNA vaccines^31^ may be a solution but requires detailed knowledge on the antigens. All ciliated stages carry antigenic epitopes, and the i‐antigen (immobilization antigen) associated with the cilia may vary between strains. Several serotypes of the i‐antigen have been identified which explain the strain‐specific immunity reported.^5^, ^6^, ^32^ In addition, screening of the parasite genome suggested that a number of other antigens (not necessarily presented at the surface of the parasite) were potentially protective.^30^

## 2 EXPERIMENTAL APPROACHES

During the latest three decades, a wide spectrum of techniques have been applied in the exploration of immune reactions in fish against *I multifiliis*.^33^, ^34^ Documentation of protective immunity is based on controlled infection and reinfection studies (challenge experiments), whereafter sampling of plasma/serum of immunized fish for in vitro analyses have been performed.^4^, ^20^, ^23^, ^32^ A basic technique applied to document the presence of a protective reaction in the fish is pathogen immobilization and/or agglutination.^5^, ^32^ Antibody reactions have been documented by ELISA and Western blotting, and histological and immunohistochemical analyses have shown the direct interaction between parasite and host factors (immunoglobulin and lymphocytes).^35^, ^36^ Gene expression analyses (quantitative QPCR) have elucidated a varied response to invasion, and establishment in specific hosts for which assays (primers and probes) have been developed to elucidate involvement of specific immune genes.^24^, ^27^ General transcriptomic analyses have been applied in order to provide an overall picture of regulation of thousands of sequences which can be compared to annotated genes associated with different physiological pathways and compartments.^16^ By analysing peptides and proteins, the proteomic approach can supplement the expression studies by presenting variations in effector molecules.^15^

## 3 IMMUNITY

### 3.1 Immune cells and tissues

Shortly after penetration of the host surface, it is possible to measure regulation of a series of immune related molecules.^33^, ^34^ Teleost surfaces are covered by mucosal tissue with associated lymphoid cell aggregations corresponding partly to the gut surface architecture in higher vertebrates including mammals.^36^ The different conglomerates of immune reactive cells in fish surfaces have been termed SALT (skin‐associated lymphoid tissue), GALT (gut‐associated lymphoid tissue), gill‐associated lymphoid tissue (GIALT), NALT (nasal‐associated lymphoid tissue) and ILT (intrabranchial lymphoid tissue).^36^, ^37^, ^38^, ^39^ However, as the lymphoid cells in these surfaces are not (apart from interbrancial lymphoid tissue) organized in discrete tissues (as in head kidney and spleen), it may be suggested to replace, in these abbreviations, ‘T’ (for tissue) with ‘C’ (for cells). Thereby, terms such as SALC, GALC, GIALC and NALC, respectively, may be preferred for specification of the cells. The immediate response to parasite penetration is associated with expression of genes encoding inflammatory cytokines and acute phase reactants,^40^, ^41^ but during the subsequent infection period, genes associated with adaptive responses involving both B and T cells are evident. Genes encoding chemokines CK‐11^42^ and cytokines IFNγ, Il‐6, IL‐10, IL‐4/13, IL‐17 and IL‐22 are upregulated shortly after exposure of the naïve fish skin to theronts reflecting that a series of innate and adaptive responses are called upon.^43^, ^44^ Transcriptomic^16^ and proteomic^15^ analyses show that the penetrating theront and the early trophont induce complicated physiological reactions at the affected site. Some of these are involved in pathogen elimination, but others contribute to re‐establishment of the injured surface structure. The developing trophont is able to feed on the proliferating epithelial cells and circumvent the host attack as illustrated by their ability to ingest also neutrophils attracted to the infection site.^14^ Following infection and even after the trophont has left the epidermis a wide range of innate immune genes stay upregulated, and their products may thereby add to the parasite hostile environment in the skin, but adaptive immune reactions appear to be major players in the protection. When immunized fish are challenged, immunoglobulin genes and Th2 associated cytokines are upregulated in connection to activation of cellular and humoral elements assisting production of antibodies.^43^

### 3.2 IgT and its role in protection

Immunoglobulin T (IgT) is a prominent antibody isotype in some teleost species which was described by Hansen et al^45^ when analysing the rainbow trout genome. In this host, at least three subclasses occur with IgT1 expressed both in mucosal and systemic lymphoid tissues, IgT2 mainly expressed at the systemic level, whereas IgT3 is generally expressed at a low systemic level.^46^ The antibody is present in both internal organs and surfaces of fish at even very early developmental stages of the trout.^47^ The dense layer of IgT in the mucous lining of naïve trout larvae may be protective—but merely partly—as yolksac larvae exposed to a high *I multifiliis* pressure become infected.^48^ Juvenile rainbow trout on the other hand (with a relatively less dense layer of IgT in the surface lining) are highly susceptible until they develop specific immunity, but the early trophont get into close contact with IgT and IgT producing cells even in naïve trout. The IgT‐*I multifiliis* interaction was documented by several studies^35^, ^36^, ^49^ and indicated to be involved in host protection as judged from the stronger binding of IgT to the parasite surface in immunized fish compared to naïve fish. IgT is also produced by Atlantic salmon,^50^ turbot,^51^ Nile tilapia,^52^ zebrafish (IgZ),^43^ stickleback and carp.^46^ However, channel catfish *Ictalurus punctatus*, which develop a strong immunity against the parasite, does not possess IgT genes and seems to rely on various forms of IgM and possibly other immune mechanisms during the combat against Ich.^53^ The local responses in this ictalurid fish host are highly developed and seems to be determined by B‐cell clones communicating between different mucosal tissues.^54^

## 4 DISCUSSION AND CONCLUSION

**Figure 6:**
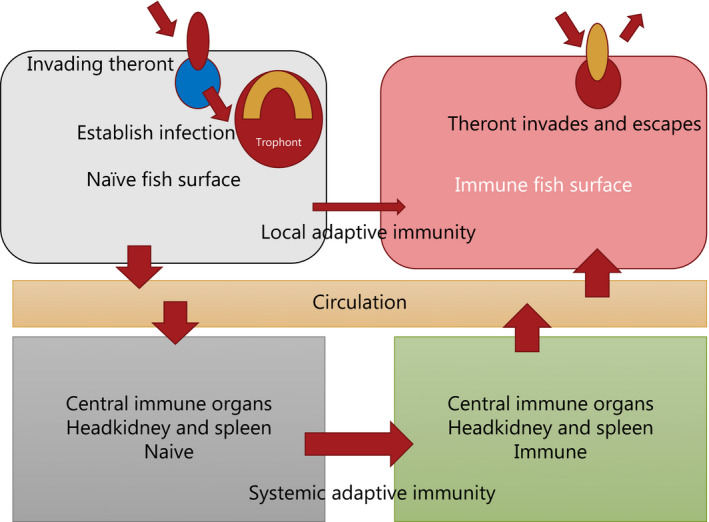
Schematic model of the immune reactions induced by the developing trophont in mucosal surfaces of the teleost fish

Development of protective immunity in fish against infections with the ciliated protozoan *I multifiliis* has been well known and recognized for more than a century. The main scientific challenge has been to describe the protective immunological mechanisms in the host and develop techniques to study the reactions. The history during the latest four decades, therefore, reflects the available methodologies which have been taken in action for the purpose. With the advent of new immunological techniques for various fish species, it has been possible in a stepwise manner to build layer on layer on our understanding of immune mechanisms. Invading *I multifiliis* theronts induce a series of physiological changes^15^ including inflammatory reactions in the affected fish surfaces, and in naïve fish, they increase with growth of the trophont (Figure 6). There is basis to suggest that messengers, antigen sampling and probably antigen presenting cells,^55^ such as dendritic cells or macrophages, internalize the antigen from the parasite in the fish surface and migrate to central immune organs, such as head kidney and spleen, for induction of adaptive specific responses against the parasitic antigens. It still has to be demonstrated if antigen presentation also occurs within the local aggregations of lymphoid tissue in the skin, gills, nostrils and buccal cavity (mucosa‐associated lymphoid cells—MALC).^35^, ^36^, ^54^ There is evidence for a local specific reaction in the fish surface, but it is connected to a central involvement of central immune organs where antigen presentation and development of adaptive immunity has been demonstrated. The wide spectrum of reactions induced in the fish skin contribute to a local environment which attract immune cells (neutrophils, dendritic cells, macrophages, nonspecific cytotoxic cells, lymphocytes and others) at the infection site. However, when the antigens are presented in the central immune organs, the inflammation is reduced and the adaptive processes take the lead.^44^ Stimulation of specific clones of lymphocytes lead to proliferation of lymphocytes which subsequently migrate to the site of action, the mucosal surfaces where the theronts seek to penetrate the host. Thus, adaptive responses in central immune organs in combination with reactions in mucosal tissues become the important events which rule the systemic immunity (Figure 6). The inflammation established by the feeding theronts in gills, fins and skin persist for a period after the parasites have left the host and the very broad spectrum of effector molecules produced by inflammatory cells may be part of the hostile microenvironment which induce escape reactions of theronts during a re‐infection.^43^ The escape reaction of theronts when host antibodies bind to their surface cilia may be the main adaptive immune mechanism,^7^, ^56^, ^57^ but it is noteworthy that the state of inflammation in the fish surface is considerably elevated. This suggests that although a series of adaptive reactions, including IgT production, are activated both at the local and the systemic level, and thus are responsible for the specific protection, innate mechanisms contribute to the hostile environment driving theronts out of the fish surface in the immune host. Complement factors may bind to specific antibodies and challenge the surface membranes of the parasites. Chemokine CK11 is known to destroy the parasite membrane directly, and SAA is likely to assist cross‐binding of surface epitopes. It is noteworthy that the cellular arm of immunity is needed for an optimal protection^10^ as a basis for the various antiparasitic effector mechanisms. All together, the numerous investigations conducted during the latest decades present a picture of a strong innate response in teleost fish which provides a fundamental protection against various diseases. By combining an elevated level of these innate effector molecules in the mucosal surfaces of fish with adaptive immune factors—here among specific immunoglobulins—it results in a satisfactory protection against *I multifiliis*.

## DISCLOSURES

None.
